# β-Keto-enol Tethered Pyridine and Thiophene: Synthesis, Crystal Structure Determination and Its Organic Immobilization on Silica for Efficient Solid-Liquid Extraction of Heavy Metals

**DOI:** 10.3390/molecules21070888

**Published:** 2016-07-07

**Authors:** Smaail Radi, Said Tighadouini, Maryse Bacquet, Stephanie Degoutin, Jean-Philippe Dacquin, Driss Eddike, Monique Tillard, Yahia N. Mabkhot

**Affiliations:** 1 Laboratoire de Chimie Appliquée et Environnement (LCAE), Faculté des Sciences, Université Mohamed I, Oujda 60 000, Morocco; tighadouinis@gmail.com; 2 Centre de l’Oriental des Sciences et Technologies de l’Eau (COSTE), Université Med I, Oujda 60 000, Morocco; 3 Unité Matériaux et Transformations (UMET) - UMR CNRS 8207 Université Lille 1, 59655 Villeneuve d’Ascq Cedex, France; maryse.bacquet@univ-lille1.fr (M.B.); stephanie.degoutin@univ-lille1.fr (S.D.); 4 Univ. Lille, CNRS, Centrale Lille, ENSCL, Univ. Artois, UMR 8181–UCCS–Unité de Catalyse et Chimie du Solide, F-59000 Lille, France; jean-philippe.dacquin@univ-lille1.fr; 5 Laboratoire de Chimie du Solide Minéral et Analytique, Faculté des Sciences, Université Mohamed I, Oujda 60 000, Morocco; eddrisse@yahoo.fr; 6 Institut Charles Gerhardt—AIME, UMR 5253, CC1502, Université de Montpellier, 2 place Eugène Bataillon, 34095 Montpellier Cedex 5, France; mtillard@univ-montp2.fr; 7 Department of Chemistry, Faculty of Science, King Saud University, P.O. Box 2455, Riyadh 11451, Saudi Arabia; yahia@ksu.edu.sa

**Keywords:** keto-enol, crystal structure, hybrid material, adsorption, heavy metals

## Abstract

Molecules bearing β-keto-enol functionality are potential candidates for coordination chemistry. Reported herein is the first synthesis and use of a novel designed ligand based on β-keto-enol group embedded with pyridine and thiophene moieties. The product was prepared in a one-step procedure by mixed Claisen condensation and was characterized by EA, *m*/*z*, FT-IR, (^1^H, ^13^C) NMR and single-crystal X-ray diffraction analysis. The new structure was grafted onto silica particles to afford a chelating matrix which was well-characterized by EA, FT-IR, solid-state ^13^C-NMR, BET, BJH, SEM and TGA. The newly prepared organic-inorganic material was used as an adsorbent for efficient solid-phase extraction (SPE) of Cu(II), Zn(II), Cd(II) and Pb(II) from aqueous solutions and showed a capture capacity of 104.12 mg·g^−1^, 98.90 mg·g^−1^, 72.02 mg·g^−1^, and 65.54 mg·g^−1^, respectively. The adsorption capacity was investigated, in a batch method, using time of contact, pH, initial concentration, kinetics (Langmuir and Freundlich models), and thermodynamic parameters (ΔG°, ΔH° and ΔS°) of the system effects.

## 1. Introduction

Molecular compounds with β-keto-enol functions have attracted great attention for several years due to their many applications in organic and inorganic chemistry [1,2,3,4,5]. In recent years, β-keto-enols ligands appear as one of the classical chelating ligands playing a significant role in coordination chemistry [6,7].

Research on β-keto-enol derivatives and their metal complexes has been stimulated by their strong complexing properties [8,9]. These types of molecules have two potential coordination sites and can: (i) behave as uni- or bidentate ligand; (ii) coordinate to the metal atom through monoionic or neutral form; and (iii) form a bridge between two metal atoms. The bidentate architecture of ligands both allows complexation and extraction with almost all metal ions [10]. Indeed, these ligands have played a significant role in extraction of metals for over a century; for example, the commercial β-keto-enol extractant showed an ability to extract the metals copper (Cu), cobalt (Co), nickel (Ni), and zinc (Zn) from ammoniacal solutions in the following order Cu > Co > Ni > Zn [11]. A fluorinated commercial β-keto-enol extractant has also been investigated and shows an extraction efficiency of transition metal ions from water and organic solvents [12]. From another study, the following order of extraction was established: Ni > Cd > Mn > Ph > Fe > Zn > Co > Pd > Cu [13].

Therefore, this class of ligands can be used successfully for heavy metal extraction and can be proposed as potential candidate for various technological applications such as hybrid organic-inorganic materials.

It has been found that the behavior of these hybrid materials used as an adsorbent is mainly dependent on the presence of β-keto-enol group embedded with heterocyclic moieties, which afford the molecule with the ability to form strong interactions with metal ions [14,15].

In continuation of our recent works in this field [14,15,16,17,18,19], herein, we report the synthesis and X-ray diffraction (XRD) structure of a new (*Z*)-3-hydroxy-3-(pyridine-2-yl)-1-(thiophen-2-yl)prop-2- en-1-on ligand based β-keto-enol. The prepared ligand was then immobilized onto silica particles and used as an adsorbent for excellent solid-phase extraction (SPE) of Cu(II), Zn(II), Cd(II) and Pb(II) from aqueous solutions. All parameters that can affect the sorption efficiency of the metal ions were studied using atomic absorption.

## 2. Results

### 2.1. Chemistry

**Scheme 1:**
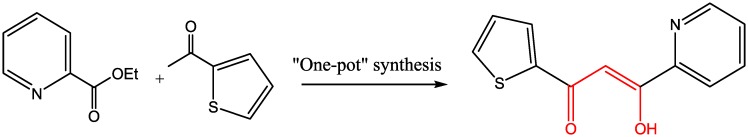
Reagents and conditions: Na, toluene, room temperature, two days, then acetic acid.

The chelating compound based on β-keto-enol group tethered pyridine and thiophene was prepared by a one-pot in situ mixed Claisen condensation as illustrated in Scheme 1.

The product was obtained using a procedure similar to that described in our recent previous work [20]. The β-keto-enol form was determined using ^1^H-NMR, showing a strong signal assigned to the =C–H group of the keto-enol form and a negligible signal attributed to the CH_2_ group of the diketone form. Traces of the keto form are detected at amounts of around 4 ppm and also observed in DEPT-135 as a very small negative signal. Good quality crystals of the β-keto-enol structure were grown from methanol solution by slow evaporation.

### 2.2. X-ray Crystal Structure Description

To obtain the crystal data and refinement parameters of (2*Z*)-3-hydroxy-3-(pyridin-2-yl)-1- (thiophen-2-yl)prop-2-en-1-one, it was subjected to X-ray diffraction intensity measurement. Single crystal data and refinement parameters are given in Table 1. CCDC 1481979 contains the supplementary crystallographic data for this paper. These data can be obtained free of charge via http://www.ccdc.cam.ac.uk/conts/retrieving.html (or from the CCDC, 12 Union Road, Cambridge CB2 1EZ, UK; Fax: +44 1223 336033; E-mail: deposit@ccdc.cam.ac.uk).

**Figure 1:**
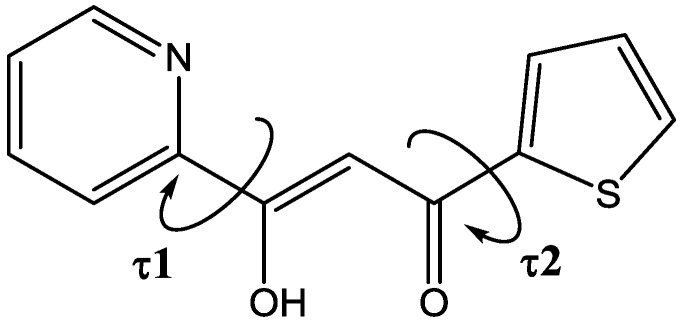
Molecular conformation.

The molecular conformation of the compound is characterized by two degree-of-freedom, which are the O2–C3–C4–C5 and O1–C1–C8–N1 torsion angles, denoted hereafter as τ1 and τ2, respectively (Figure 1).

**Figure 2:**
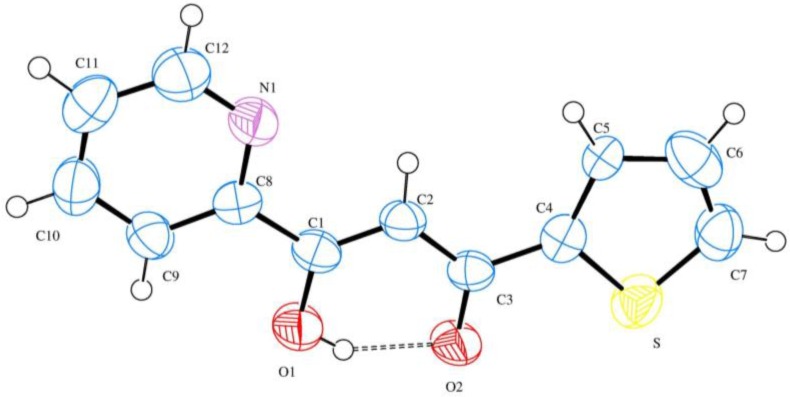
X-ray diffraction (XRD) of the target product.

The values of −177.5(5) and 177.0(5), respectively, indicate small deviations of thiophene and pyridine groups from the plane formed by ketone and enol groups. Normally, two isomers only differing by the relative positions of ketone and enol groups would be expected in this reaction. Nevertheless, in present conditions, the reaction leads to formation in 95% yield of the isomer shown in Figure 2. The same observation was noted for our other keto-enol derivatives recently postponed [21,22,23,24,25].

The compound shows an intramolecular O2∙∙∙H-O1 hydrogen bond of 2.488 Å and 148.30° involving ketone and hydroxyl groups. Moreover, each molecule is linked to four neighboring molecules via weak hydrogen bonding of 2.589 Å and 2.833 Å at H5∙∙∙C5 and H7∙∙∙O1 atomic pairs.

### 2.3. Immobilization on Silica

#### 2.3.1. Linker Synthesis

**Scheme 2:**
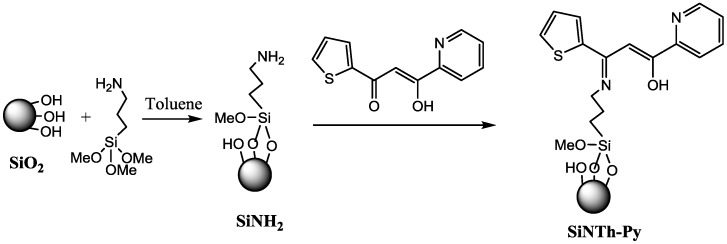
The synthesis route of modified chelating material.

The synthetic procedure for the new chelating material can be summarized in Scheme 2. The preparation involves reaction of the activated silica gel with 3-aminopropyltrimethoxysilane in toluene to form the amino groups attached to the silica surface [26]. These NH_2_-groups onto the silica surface are then reacted with (*Z*)-3-hydroxy-3-(pyridine-2-yl)-1-(thiophen-2-yl)prop-2-en-1-on under gentle conditions (reflux, 24 h), using anhydrous methanol as solvent to form the new chelating sorbent SiNTh-Py.

#### 2.3.2. Characterization

The C and N contents (%C = 4.46 and %N = 1.66) in SiNH_2_ are a sign of successful aminopropylation reaction. The increase in %C and %N contents in SiNTh-Py (%C = 11.34 and %N = 3.72) indicates that the (*Z*)-3-hydroxy-3-(pyridine-2-yl)-1-(thiophen-2-yl)prop-2-en-1-on is attached to SiNH_2_.

**Figure 3:**
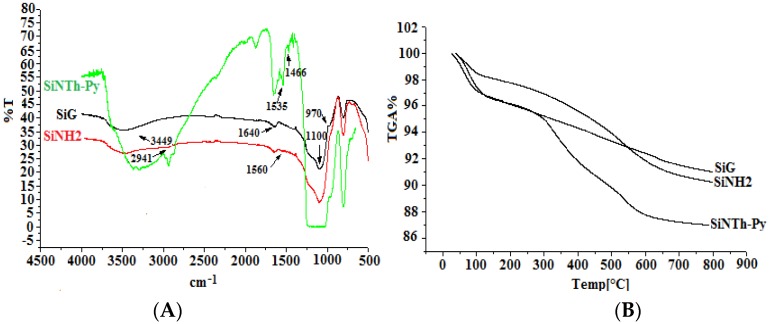
(**A**) FT-IR Spectra; (**B**) Thermogravimetric curves of free silica SiG, SiNH_2_ and SiNTh-Py.

The FT-IR of SiNH_2_ (Figure 3A) exhibits ν_C–H_ and ν_NH2_ bands at 2941 cm^−1^ and 1560 cm^−1^, respectively, from silylating 3-aminopropyltremethoxysilane, which are absent in the spectrum of unmodified silica gel. In the FT-IR spectrum of SiNTh-Py, obtained after reaction with β-keto-enol, the bands ν_C=C_ and ν_C=N_ at 1466 cm^−1^ and 1535 cm^−1^, respectively, demonstrate the successful immobilization of (*Z*)-3-hydroxy-3-(pyridine-2-yl)-1-(thiophen-2-yl)prop-2-en-1-on on SiNH_2_.

The Thermogravimetric curves TGA curve recorded for the starting silica shows only one mass change in the range of 25 °C–110 °C. This mass loss corresponds to the loss of the remaining absorbed water. The TGA curve of free silica, SiNH_2_, and SiNTh-Py are represented in Figure 3B. SiNTh-Py shows two stages of weight loss, the first one is similar to that of pure silica with 3.04% weight loss, and is followed by 10.00% weight loss around 110 °C–800 °C corresponding to the loss of the organic groups. This observation shows that the organic part is immobilized on the silica.

**Figure 4:**
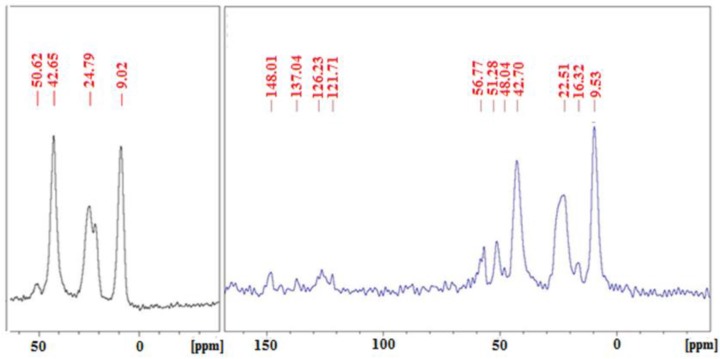
^13^C-NMR spectra of (SiNH_2_) and (SiNTh-Py).

The solid-state ^13^C-NMR spectrum is shown in Figure 4. The signals observed for 3-aminopropyl-silica SiNH_2_ at δ= 9.02, 24.79 and 42.62 ppm have been assigned to the propyl carbon Si–CH_2_, –CH_2_–, and N–CH_2_, respectively. The signal at 50.62 ppm is assigned to methoxy group –OCH_3_ not substituted as confirmed by microanalysis. Other signals at 16.32, 48.04, 56.77, 121.71, 126.23, 137.04, and 148.01 ppm correspond to specific carbons atoms in (*Z*)-3-hydroxy-3-(pyridine-2-yl)-1-(thiophen-2-yl)prop-2-en-1-on moiety.

**Figure 5:**
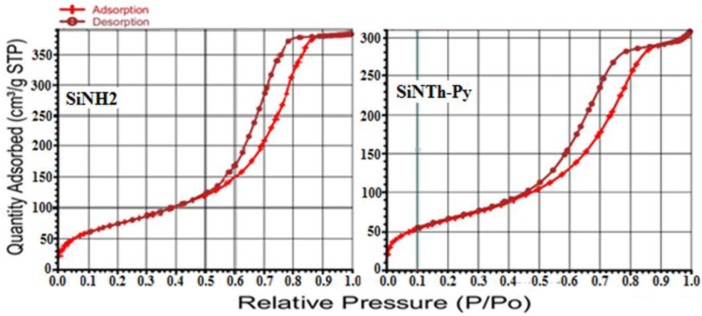
Nitrogen adsorption-desorption isotherm plots of SiNH_2_ and SiNTh-Py.

The specific surface areas (Figure 5) of free silica, SiNH_2_, and SiNTh-Py are 305.21, 283.08, and 229.59 m^2^/g respectively, and the pore volumes of these materials are 0.77, 0.69, and 0.59 cm^3^/g, respectively. Therefore, a decrease in the specific surface areas and pore volumes are due to the functionalization of silica.

#### 2.3.3. Solid-Liquid Adsorption of Metal Ions

##### Effect of pH and Stirring Time

**Figure 6:**
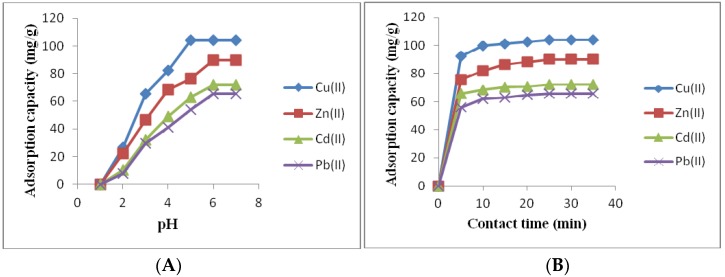
(**A**) Effect of pH; (**B**) Effect of shaking time on the adsorption capacity of Cu(II), Zn(II), Cd(II) and Pb(II).

The effect of solution pH on the removal of Cu(II), Zn(II), Cd(II), and Pb(II) by SiNTh-Py is shown in Figure 6A. Metal ion removal by the adsorbent is increased when there is an increase in the pH of the solution. The maximal removal of Cu(II) was obtained at pH = 5, but it occurs at pH = 6 for Zn(II), Cd(II) and Pb(II).

The contact time (Figure 6B) reveals that the equilibrium is reached after only 25 min. This result indicates that the SiNTh-Py adsorbent has rapid adsorption kinetics. Therefore, it is suitable for an application in flow system as used in the preconcentration of trace metal ions.

Furthermore, the adsorbent presents higher adsorption capacity toward Cu(II) compared to the other metals under study. This is mainly dependent on several factors such as the nature, the charge, and the size of metal ions, and the affinity of donor atoms towards metals. This affinity towards Cu(II) allowing the extraction of 104 mg/g must be underlined, whereas the adsorption capacity of the SiO_2_ matrix was only 1 mg/g [27].

In order to investigate the mechanism of adsorption, kinetic parameters were evaluated using pseudo-first order [28] and pseudo-second order [29] models (Table 2). It is evident from Table 2 that, for all metals under study, values of the regression coefficient are much higher from pseudo-second order model than from pseudo-first order kinetic model. Furthermore, theoretical and experimental values of q_e_ are close for pseudo-second order kinetics; this indicates the pseudo-second order model fits well with the experimental adsorption data.

##### Adsorption Isotherms

The experimental data have been tested within two isotherm models. The first one is the Langmuir isotherm model [30] that describes the monolayer coverage adsorption and homogeneous surface. The second model is the Freundlich isotherm model [31] adapted to the description of the multilayer sorption and heterogeneous surface.

The Langmuir and Freundlich isotherm parameters for adsorption of Cu(II), Zn(II), Cd(II), and Pb(II) are given in Table 3. Comparison of the R^2^ values shows that the experimental data are quite well-fitted using the Langmuir isotherm model.

##### Thermodynamics Adsorption

Energetic changes associated with the removal of Cu(II), Zn(II), Cd(II), and Pb(II) onto SiNTh-Py can be evaluated with the help of thermodynamic parameters (ΔG°, ΔH° and ΔS°) [32,33,34]. The results are given in Table 4. The negative values of ΔG° indicate the feasible and spontaneous nature of adsorption. The positive values of enthalpy ΔH° reveal that adsorption is endothermic. The positive values of ΔS° suggest a more random organization at the solid/solution interface.

##### Competitive Adsorption

**Figure 7:**
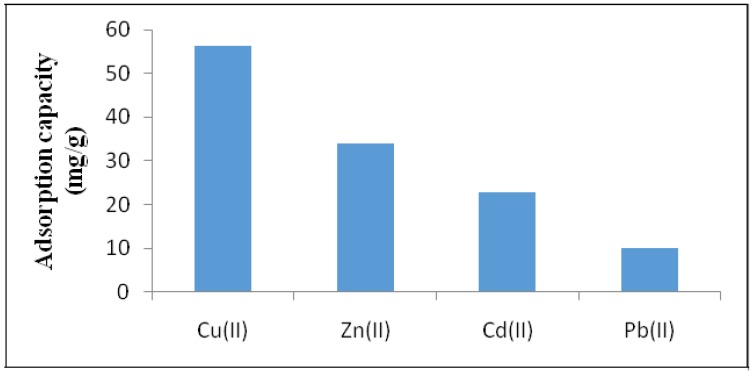
Effect of foreign metal ions on the extraction of Cu(II) with SiNTh-Py (shaking time 60 min, pH = 6, V = 10 mL, m = 10 mg of SiNTh-Py and: 140 mg/L of each metals).

The competitive adsorption experiment was carried out for Cu(II), Zn(II), Pb(II), and Cd(II) quaternary systems using an aqueous solution containing 140 mg/L of each metal ion. Figure 7 shows the adsorption capacity of metal ions in the quaternary systems. It is obvious that SiNTh-Py displays an excellent adsorption for Cu(II). However, the extraction seems to decrease with regard to the value obtained in the individual adsorption experiments, indicating a competitive complexation with other ions.

Thus, the SiNTh-Py shows promising potential to be a good adsorbent, particularly for the removal of Cu(II) from aqueous solutions containing competing ions.

##### Comparison with Alternative Adsorbents

Compared to several sorbents recently described in the literature (Table 5), the adsorbent prepared in the present work exhibits a higher adsorption capacity, especially for Cu(II). This efficiency is mainly due to the affinity of the ligand donor atoms towards this metal.

## 3. Materials and Methods

### 3.1. General Information

All solvents and other chemicals (purity >99.5%, Aldrich, St. Louis, MO, USA) were of analytical grade and used without further purification. Silica gel (E. Merck, Darmstadt, Germany)—with particle size in the range of 70–230 mesh, median pore diameter 60 Å—was activated before use by heating it at 160 °C for 24 h. The silylating agent 3-aminopropyltrimethoxtsilane (Janssen Chimica, Geel, Belgium) was used without purification. Oxford Diffraction Xcalibur Sapphire3 Gemini ultra CCD diffractometer was used to collect the X-ray diffracted intensities from a parallelepiped selected single crystal. All metal ions that were determined by atomic adsorption measurements were performed using a Spectra Varian A.A. 400 spectrophotometer (Oujda, Morocco). The pH value was controlled by a pH 2006, J. P. Selecta s. a. (Barcelona, Span). Elemental analyses were performed by the Microanalysis Centre Service (CNRS, Lille, France). FT-IR spectra were obtained with a Perkin Elmer System 2000 instrument (Oujda, Morocco). SEM images were obtained on an FEI-Quanta 200 (Lille, France). The mass loss determinations were performed in 90:10 oxygen/nitrogen atmospheres on a Perkin Elmer Diamond TG/DTA, at a heating rate of 10 °C·min^−1^ (Blois, France). The ^13^C-NMR spectrum of the solid state was obtained with a CP MAX CXP 300 MHz instrument (Lille, France). The specific area of the modified silica was determined by using the BET equation. The nitrogen adsorption-desorption was obtained by means of a Thermoquest Sorpsomatic 1990 analyzer (Lille, France), after the material had been purged in a stream of dry nitrogen. Molecular weights were determined on a JEOL JMS DX-300 Mass Spectrometer (CNRST, Rabat, Morocco).

### 3.2. Procedure for the Synthesis of β-Keto-enol Heterocycle

Metallic sodium (15.21 mmol) was slowly added the pyridine carboxylate (12.01 mmol) in 25 mL of toluene. Then, thiophene methyl ketone (12.01 mmol) in 10 mL of toluene was added at 0 °C and the mixture was stirred at room temperature for 2 days. The resulting precipitate was filtered, washed and dissolved in water to be neutralized with acetic acid to pH 5. The extracted organic layer was dried and concentrated in vacuum. The obtained residue was filtered through silica using CH_2_Cl_2_/MeOH as eluant to give the desired product as a white solid in 38% yield. The β-keto-enol form was recrystallized from methanol (95%) to obtain the target compound which was confirmed by FT-IR, ^1^H-NMR, ^13^C-NMR, elemental analysis, and mass spectroscopy.

*(Z)-3-Hydroxy-3-(pyridine-2-yl)-1-(thiophen-2-yl)prop-2-en-1-on*: colorless crystals; yield: 38%; m.p. 94 °C–96 °C; *R_f_* = 0.13 (CH_2_Cl_2_/MeOH, 9/1)/silica. IR (KBr, cm^−1^): ν(OH) = 3426; ν(C=O) = 1622; ν (enolic C=C) = 1514; ^1^H-NMR (DMSO-*d*_6_): δ 7.275 (m, 1H, Py-Hε); 7.396 (s, 1H, enol, C–H); 7.637 (t, 1H, Py-Hδ); 8.007 (t, 1H, Py-Hγ); 8.061 (t, 1H, Th-Hβ); 8.065 (d, 1H, Th-Hγ); 8.119 (d, 1H, Th-Hα); 8.749 (d, 2H, Py-Hα); ^13^C-NMR (DMSO-*d*_6_): δ 93.968 (1C, enol, C–H); 122.100 (1C, Py-Cδ); 127.504 (1C, Th-Cβ); 128.589 (1C, Py-Cβ); 129.375 (1C, Th-Hγ); 133.039 (1C, Th-Cα); 135.677 (1C, Py-Cγ); 136.064 (1C, Th-Cε); 138.252 (1C, Py-Cε); 150.228 (1C, Py-Cα); 178.155 (1C, C=O); 183.895 (1C; C–OH); M.S: *m*/*z*, 232.02 [M + H]^+^. Anal. Calcd. For C_12_H_9_NO_2_S: C, 62.32; H, 3.92; N, 6.06; S, 13.86. Found: C, 61.79; H, 3.96; N, 6.09; S, 14.03.

### 3.3. Synthesis of 3-Aminopropylsilica (SiNH_2_)

The 3-aminopropylsilica (SiNH_2_) material was prepared using our recently reported method [14,15,16,17,18,19].

### 3.4. Synthesis of Pyridine-enol-imine-thiophene-Substituted Silica (SiNTh-Py)

Five grams of SiNH_2_ was treated with 3 g of synthesized ligand, dissolved in 50 mL of dry methanol. The mixture was refluxed for 24 h. The solid was filtered, dried and then Soxhlet extracted with acetonitrile, methanol and dichloromethane for 12 h. The product was then dried under vacuum at 70 °C over 24 h.

### 3.5. Batch Method

Effects of pH of the solution and contact time on the sorption of metal ions were evaluated using a batch method. A suspension of 10 mg of adsorbent in 10 mL of metal solution at optimum concentration (140 mg/g) of each metal ion was mechanically stirred at room temperature. In addition, this method was used to study the adsorption isotherm, adsorption thermodynamics, and competitive adsorption. After extraction, the residual metal concentration of the supernatant was determined by atomic absorption measurements. All experiments were performed in duplicate.

### 3.6. X-ray Diffraction Analysis

Xcalibur, Sapphire3, Gemini CCD plate diffractometer was used to perform X-ray analysis on the parallelepiped colourless sample. MoKα radiation (λ = 0.71073 Å) and ω scan were employed in the data collection. Data collection, cell refinement and data reduction were carried out with CrysAlis 171 Oxford Diffraction, 2009 software. In this analysis, all the crystallographic data were collected at room temperature. The SHELXS-97 program [40] was used to solve the structure with direct methods. Refinements of the structure on F^2^ were done using full-matrix least-squares techniques with SHELXL-2013 software [40]. Anisotropic refinements were applied on all non-hydrogen atoms. All C-bound hydrogen atoms were inserted at their calculated positions and then refined using a riding model. The hydrogen isotropic displacement parameters are set to 1.2 (or 1.5 for methyl groups) times the equivalent isotropic U values of the parent carbon atoms. Figures were prepared using ortep3 [41] and mercury 3.8 [42] programs. Complete crystallographic data for the studied compound have been deposited at the Cambridge Crystallographic Data Centre with CCDC deposition number of 1481979.

## 4. Conclusions

Based on the experimental results, it can be concluded that a new highly chelating β-keto-enol bis-heterocyclic ligand has been synthesized and its XRD single crystal structure determined. A novel organic-inorganic hybrid material, supporting the new ligand receptor, has been successfully prepared via a simple heterogeneous procedure, and the surface is well characterized. The functionalized material displays an excellent adsorption capacity towards Cu(II), Zn(II), Cd(II), and Pb(II). The maximum values for adsorption were reached in only 25 min, suggesting rapid coordination. The adsorption kinetics fit into the pseudo-second-order model, which reveals a homogeneous character. The thermodynamic parameters are in agreement with an endothermic and spontaneous process. The competitive adsorption proves the efficiency of this new organic-inorganic hybrid material for removing heavy metals, especially Cu(II), from aqueous solutions.

## Figures and Tables

**Table 1:** Crystal data and refinement parameters for C_12_H_9_NO_2_S.

CCDC Deposition Number	1481979
Molecular Formula	C_12_H_9_NO_2_S
Molecular Weight	231.26
Crystal System	orthorhombic
Space Group	P n a 2_1_
*a* (Å)	15.2526 (9)
*b* (Å)	18.3543 (10)
*c* (Å)	3.8806 (3)
α (°)	90
β (°)	90
γ (°)	90
*V* (Å^3^)	1086.38 (11)
*Z*	4
*D*calc (g·cm^−3^)	1.414
Crystal Dimension (mm)	0.35 × 0.13 × 0.12
μ (mm^−1^)	0.280
*T*min/*T*max	0.908/0.967
Measured Reflections	4873
	−17, 17
Indices Range (*h*, *k*, *l*)	−21, 19
	−5, 3
θ Limit (°)	1.736–27.718
Unique Reflections	1801
Observed Reflections (*I* > 2σ(*I*))	1402
Parameters	151
Goodness of Fit on *F*^2^	1155
*R*_1_, *wR*_2_ (*I* > 2σ(*I*))	0.0612, 0.1578

**Table 2:** Kinetics of heavy metals removal onto SiNTh-Py (at pH = 6, V = 10 mL, m = 10 mg of SiNTh-Py and optimum concentration: 140 mg/L in each case).

Parameters	Metals			
	Cu(II)	Zn(II)	Cd(II)	Pb(II)
q_e(exp)_ (mg/g)	104.12	98.90	72.02	65.54
Pseudo-first-order				
q_e_ (mg/g)	19.78	33.28	10.07	22.02
k_1_ (min^−1^)	0.128	0.153	0.102	0.169
R^2^	0.960	0.960	0.997	0.945
Pseudo-second-order				
q_e_ (mg/g)	104.16	90.90	71.94	66.22
k_2_ (g/mg·min)	3.76 × 10^−3^	17.28 × 10^−3^	23.29 × 10^−3^	29.61 × 10^−3^
R^2^	0.993	0.997	0.999	0.998

**Table 3:** Adsorption isotherm parameters for the removal of heavy metals onto SiNTh-Py (shaking time 60 min, pH = 6, V = 10 mL, m = 10 mg of SiNTh-Py, optimum concentration: 140 mg/L in each case).

Metal	Langmuir Isotherm Model			Freundlich Isotherm Model		
	q (mg/g)	K_L_ (L/mg)	R^2^	K_F_ (mg/g)	*n*	R^2^
Cu(II)	106.38	0.425	0.998	39.87	04.23	0.774
Zn(II)	96.15	0.138	0.995	16.74	02.47	0.923
Cd(II)	78.12	0.141	0.994	08.54	01.90	0.941
Pb(II)	67.56	0.328	0.998	26.58	04.59	0.829

**Table 4:** Adsorption models used in this work and their parameters (shaking time 60 min, pH = 6, V = 10 mL, m = 10 mg of SiNTh-Py at optimum concentration: 140 mg/L in each case).

Metal	ΔH° (kJ·mol^−1^)	ΔS° (Jk^−1^·mol^−1^)	T (°C) ± 1 °C	ΔG° (kJ·mol^−1^)
Cu(II)	19.55	70.23	25	−1.45
			35	−2.15
			45	−2.89
Zn(II)	10.23	36.16	25	−0.58
			35	−0.94
			45	−1.30
Cd(II)	09.27	31.45	25	−0.13
			35	−0.44
			45	−0.76
Pb(II)	22.05	78.95	25	−1.56
			35	−2.35
			45	−3.14

**Table 5:** Cu^2+^ adsorption performances of SiNTh-Py compared with that of some recently reported sorbents.

Support: Silica Gel/Ligand	Ref.	Adsorption Capacity (mg/g)
((*Z*)-3-hydroxy-3-(pyridine-2-yl)-1-(thiophen-2-yl)prop-2-en-1-on	This work	104.12
1-(Furan-2-yl) imine	[19]	77.48
EDTA	[35]	85.75
Pentane-1,2-dicarboxylic acid	[36]	38.00
Schiff base tailed silatranes	[37]	13.15
Stearic acid	[38]	63.00
4-amino-3-hydrazino-5-mercapto-1,2,4-triazole	[39]	05.02

## References

[1] Okamura H., Hirayama N., Morita K., Shimojo K., Naganawa H. (2010). Synergistic effect of 18-crown-6 derivatives on chelate extraction of lanthanoids(III) into an ionic liquid with 2-thenoyltrifluoroacetone. Anal. Sic..

[2] Wilson J.J., Lipparad S.J. (2012). In vitro anticancer activity of *cis*-diammineplatinum(II) complexes with β-diketonate leaving group ligands. J. Med. Chem..

[3] Xu D.F., Shen Z.H., Shi Y., He Q., Xia Q.C. (2010). Synthesis, characterization, crystal structure, and biological activity of the copper complex. Russ. J. Coord. Chem..

[4] Almeida J.C., Marzano I.M., Silva de Paula F.C., Pivatto M., Lopes N.P., de Souza P.C., Pavan F.R., Forminga A.L.B., Pereira-Maia E.C., Guerra W. (2014). Complexes of platinum and palladium with β-diketones and DMSO: Synthesis, characterization, molecular modeling, and biological studies. J. Mol. Struct..

[5] El-Sonbati A.Z., Diab M.A., Belal A.A., Morgan S.M. (2012). Supramolecular structure and spectral studies on mixed-ligand complexes derived from β-diketone with azodye rhodanine derivatives. Spectrochim. Acta A.

[6] Pettinari C., Marchetti F., Drozdov A. (2003). β-Diketones and related ligands. Compr. Coord. Chem. II.

[7] Aromi G., Gamez P., Reedijk J. (2008). Poly β-diketones: Prime ligands to generate supramolecular metalloclusters. Coord. Chem. Rev..

[8] Sheikh J., Juneja H., Ingle V., Ali P., BenHadda T. (2013). Synthesis and in vitro biology of Co(II), Ni(II), Cu(II) and Zinc(II) complexes of functionalized β-diketone bearing energy buried potential antibacterial and antiviral *O*,*O* pharmacophore sites. J. Saudi Chim. Soc..

[9] Huang C.-H., Wang K. (2010). Rare Earth Coordination Chemistry.

[10] Furniss B.S., Hannaford A.J., Smith P.W.G., Tatchell A.R. (1989). Vogel’s Textbook of Practical Organic Chemistry.

[11] Przeszlakowski S., Wydra H. (1982). Extraction of nickel, cobalt and other metals (Cu, Zn, Fe(III)) with a commercial β-diketone extractant. Hydrometallurgy.

[12] Nakashima K., Maruyama T., Kubota F., Goto M. (2009). Metal extraction from water and organic solvents into fluorous solvents by fluorinated beta-diketone and its application to the colorimetric analysis of metal ions. Anal. Sci..

[13] Jyothi J., Rao G.N. (1988). Solvent extraction of metals with a commercial fluorinated β-diketone (LIX51) extractant. Inorg. Anal..

[14] Radi S., Tighadouini S., Bacquet M., Degoutin S., Revel B., Zaghrioui M. (2015). Quantitative removal of Zn(II) from aqueous solution and natural water using new silica-immobilized ketoenol-pyridine receptor. J. Environ. Chem. Eng..

[15] Radi S., Tighadouini S., El Massaoudi M., Bacquet M., Degoutin S., Revel B., Mabkhot Y.N. (2015). Thermodynamics and Kinetics of Heavy Metals Adsorption on Silica Particles Chemically Modified by Conjugated β-Ketoenol Furan. J. Chem. Eng. Data.

[16] Radi S., Toubi Y., Bacquet M., Degoutin S., Mabkhot Y.N., Garcia Y. (2016). An inorganic-organic hybrid material made of a silica-immobilized Schiff base receptor and its preliminary use in heavy metal removal. ACS Adv..

[17] Radi S., Tighadouini S., Bacquet M., Zaghrioui M. (2016). New adsorbent material based on nitrothiophene-functionalized silica particles for aqueous heavy metals removal. J. Sulfur Chem..

[18] Radi S., Attayibat A., El Massaoudi M., Bacquet M., Jodeh S., Warad I., Al-Showiman S.S., Mabkhot Y.N. (2015). C,N-Bipyrazole Receptor Grafted onto a Porous Silica Surface as a Novel Adsorbent Based Polymer Hybrid. Talanta.

[19] Tighadouini S., Radi S., Bacquet M., Dacquin J.-P., Mabkhot Y.N., Jodeh S., Warad I., Zaghrioui M. (2015). Synthesis of 1-(Furan-2-yl) imine Functionalized Silica as a Chelating Sorbent and its Preliminary Use in Metal Ion Adsorption. Sep. Sci. Technol..

[20] Radi S., Tighadouini S., Feron O., Riant O., Bouakka M., Benabbes R., Mabkhot Y.N. (2015). Synthesis of Novel β-Keto-enol Derivatives Tethered Pyrazole, Pyridine and Furan as New Potential Antifungal and Anti-Breast Cancer Agents. Molecules.

[21] Radi S., Tighadouini S., Ben Hadda T., Akkurt M., Özdemir N., Sirajuddin M., Mabkhot Y.N. (2016). Crystal structure of (2*Z*)-3-hydroxy-1-(1,5-dimethyl-1Hpyrazol-3-yl)but-2-en-1-one. Z. Kristallogr. NCS.

[22] Radi S., Tighadouini S., Eddike D., Tillard M., Mabkhot Y.N. (2016). Crystal structure of (*Z*)-1-(1,5-dimethyl-1*H*-pyrazol-3-yl)-3-hydroxy-3-(4-methoxyphenyl)prop-2-en-1-one. Z. Kristallogr. NCS.

[23] Radi S., Tighadouini S., Eddike D., Tillard M., Mabkhot Y.N. (2016). Crystal structure of (*Z*)-1-(1,5-dimethyl-1*H*-pyrazol-3-yl)-3-(4-ethoxyphenyl)-3-hydroxyprop-2-en-1-one. Z. Kristallogr. NCS.

[24] Radi S., Tighadouini S., Eddike D., Tillard M., Mabkhot Y.N. (2016). Crystal structure of (*Z*)-1-(1,5-dimethyl-1*H*-pyrazol-3-yl)-3-hydroxy-3-phenylprop-2-en1–1-one. Z. Kristallogr. NCS.

[25] Radi S., Tighadouini S., Eddike D., Tillard M., Mabkhot Y.N. (2016). Crystal structure of (*Z*)-1-(1,5-dimethyl-1*H*-pyrazol-3-yl)-3-hydroxy-3-(p-toly)prop-2-en-1-one. Z. Kristallogr. NCS.

[26] Qu R., Wang M., Sun C., Zhang Y., Ji C., Chen H., Meng Y., Yin P. (2008). Chemical modification of silica-gel with hydroxyl-oramino-terminated polyamine for adsorption of Au(III). Appl. Surf. Sci..

[27] Radi S., Basbas N., Tighadouini S., Bacquet M., Degoutin S., Cazier F. (2013). New amine-modified silicas: Synthesis, characterization and its use in the Cu(II) removalfrom aqueous solutions. Prog. Nanotechnol. Nanomater..

[28] Lagergren S. (1898). Theorie der sogenannten adsorption geloster stoffe, kungliga svenska vetenskapsakademiens. Handlingar.

[29] Ho Y.S., McKay G. (1999). Pseudo-second order model for sorption process. Process. Biochem..

[30] Langmuir I. (1916). The constitution and fundamental properties of solids and liquids. J. Am. Chem. Soc..

[31] Freundlich H.M.F. (1906). Über die adsorption in lösungen. Z. Phys. Chem..

[32] Sari A., Tuzen M., Uluözlü Ö.D., Soylak M. (2007). Biosorption of Pb(II) and Ni(II) from aqueous solution by lichen (*Cladonia furcata*) biomass. Biochem. Eng. J..

[33] Fan T., Liu Y., Feng B., Zeng G., Yang C., Zhou M., Zhou H., Tan Z., Wang X. (2008). Biosorption of cadmium(II), zinc(II) and lead(II) by *Penicillium simplicissimum*: Isotherm, kinetics and thermodynamics. J. Hazard. Mater..

[34] Ucun H., Bayhan Y.K., Kaya Y. (2008). Kinetic and thermodynamic studies of the biosorption of Cr(VI) by Pinus sylvestris Linn. J. Hazard. Mater..

[35] Ezzedine Z., Batonneau-Gener I., Pouilloux Y., Pouilloux H., Hamad H., Saad Z., Kazpard V. (2015). Divalent heavy metals adsorption onto different types of EDTA-modified mesoporous materials: Effectiveness and complexation rate. Micropor. Mesopor. Mater..

[36] Parambadath S., Mathew A., Park S.S., Ha C.S. (2015). Pentane-1,2-dicarboxylic acid functionalized spherical MCM-41: A simple and highly selective heterogeneous ligand for the adsorption of Fe^3+^ from aqueous solutions. J. Environ. Chem. Eng..

[37] Mutneja R., Singh R., Kaur V., Wagler J., Felsc S., Kroke E. (2016). Schiff base tailed silatranes for the fabrication of functionalized silica based magnetic nano-cores possessing active sites for the adsorption of copper ions. New J. Chem..

[38] Zhu Z. (2015). Preparation and characterization of functionalized silica spheres for removal of Cu(II), Pb(II), Cr(VI) and Cd(II) from aqueous solutions. RSC Adv..

[39] Wondracek M.H.P., Jorgetto A.O., Silva A.C.P., Ivassechen J.D.R., Schneider J.F., Saeki M.J., Pedrosa V.A., Yoshito W.K., Colauto F., Ortiz W.A. (2016). Synthesis of mesoporous silica-coated magnetic nanoparticles modified with 4-amino-3-hydrazino-5-mercapto-1,2,4-triazoleand its application as Cu(II) adsorbent from aqueous samples. Appl. Surf. Sci..

[40] Sheldrick G.M. (2008). A short history of SHELX. Acta Crystallogr. Sect. A Found. Crystallogr..

[41] Farrugia L.J. (2012). WinGX and ORTEP for Windows: An update. J. Appl. Crystallogr..

[42] Macrae C.F., Edgington P.R., McCabe P., Pidcock E., Shields G.P., Taylor R., Towler V.D.S. (2006). Mercury: Visualization and analysis of crystal structures. J. Appl. Crystallogr..

